# TFDP3 regulates the apoptosis and autophagy in breast cancer cell line MDA-MB-231

**DOI:** 10.1371/journal.pone.0203833

**Published:** 2018-09-20

**Authors:** Ling-yu Ding, Ming Chu, Yun-shen Jiao, Qi Hao, Peng Xiao, Huan-huan Li, Qi Guo, Yue-dan Wang

**Affiliations:** 1 Department of Immunology, School of Basic Medical Science, Peking University, Beijing, China; 2 Key Laboratory of Medical Immunology, Ministry of Health, Beijing, China; 3 Department of Medical Genetics, School of Basic Medical Science, Peking University, Beijing, China; 4 State Key Laboratory of Genetic Engineering, School of Life Science, Collaborative Innovation Center of Genetics and Development, Fudan University, Shanghai, China; 5 School of Basic Medical Science, Peking University, Beijing, China; University of South Alabama Mitchell Cancer Institute, UNITED STATES

## Abstract

Cancer/testis antigen TFDP3 belongs to the transcription factor DP(TFDP) family. It can bind to E2F family molecules to form a heterodimeric transcription factor E2F/TFDP complex. The complex is an important regulatory activator of cell cycle, involved in the regulation of cell proliferation, differentiation, apoptosis and other important physiological activities. In addition, TFDP3 has also been found to be a tumor-associated antigen that only expresses in malignant tumor tissue and normal testicular tissue; Thus, it is closely related to tumor occurrence and development. In this study, our group investigated the expression of TFDP3 in mononuclear cell samples from a variety of tissue-derived malignant tumors, breast cancer and benign breast lesions. The results show that TFDP3 is expressed in the malignant form of various tissues. Moreover, our recent research had focused on the ability of TFDP3 to influence the drug resistance and apoptosis of tumor cells. To further clarify the mechanisms involved in tumor resistance, this study also examined the expression of TFDP3 and tumor cell autophagy regulation; Autophagy helps cells cope with metabolic stress (such as in cases of malnutrition, growth factor depletion, hypoxia or hypoxia) removes erroneously folded proteins or defective organelles to prevent the accumulation of abnormal proteins; and removes intracellular pathogens. Our results showed that TFDP3 expression can induce autophagy by up-regulating the expression of autophagic key protein LC3(MAP1LC3) and increasing the number of autophagosomes during chemotherapy of malignant tumors. Then, DNA and organelles damage caused by the chemotherapy medicine are repaired. Thus, TFDP3 contributes toward tumor cell resistance. When siRNA inhibits TFDP3 expression, it can reduce cell autophagy, improving the sensitivity of tumor cells to chemotherapy drugs.

## Introduction

Malignant breast cancer is a lethal disease, usually characterized by aggressive phenotype, increased risk recurrence and poor prognosis. Although surgery, chemotherapy, radiotherapy and immunotherapy are considered contemporary treatment options, it still poses a serious threat to human life and health. Among the challenges treating this disease, tumor recurrence and drug resistance are the most common and are extremely difficult to tackle [1].

A Cancer/testis antigen, TFDP3, belongs to the transcription factor DP (TFDP) family. It can bind to E2F family molecules to form a heterodimeric transcription factor E2F / TFDP complex. As an important regulatory activator of cell cycle, the complex is involved in the regulation of cell proliferation, differentiation, apoptosis and other important physiological activities [2–5]. In addition, TFDP3 is a tumor-associated antigen only expressed in malignant tumor tissues and normal testicular tissue. Our former research demonstrated that TFDP3 is closely related to tumor occurrence and development. We previously reported that breast cancer with high expression of TFDP3 was much more invasive and this trait could be reversed once TFDP3 was knockdown in breast cancer cell line MDA-MB-231[6]. And in a large sample size of breast cancer microarray analysis, the expression of TFDP3 was related with HER2-overexpression subtype of breast cancer, it indicated that TFDP3 may play an important role in the diagnosis and drug-resistant of HER2-overexpression breast cancer.

In this study, we investigated the expression of TFDP3 in a variety of breast cancer cell lines and its subcellular localization. With the deployment of siRNA interference technology in vitro, one can recover the sensitivity of chemotherapy drugs in drug-resistant tumor cells by downregulating the expression of TFDP3. To further clarify the mechanism by which TFDP3 promotes tumor growth, we also studied the apoptosis rate and autophagy regulation of the TFDP3-knockdown breast cancer cell. Autophagy can help cells cope with metabolic stress like malnutrition and hypoxia, remove erroneously folded proteins or defective organelles to prevent the accumulation of abnormal proteins, and remove intracellular pathogens [7–10].

Our results show that overexpression of TFDP3 can induce autophagy by up-regulating the expression of autophagy marker light chain 3(LC3, MAP1LC3) and increasing the number of autophagosomes during chemotherapy of malignant tumors. Then, any DNA damage and cytoplasmic organelle injury caused by the chemotherapy medicine are repaired due to the occurrence of autophagy. Thus, TFDP3 is involved in the production of tumor cell resistance and, when siRNA inhibits TFDP3 expression, it can reduce cell autophagy so then improve the sensitivity of tumor cells to chemotherapy drugs.

## Materials and methods

### Tissue microarray immunohistochemical evaluation

For deparaffinization, Tissue microarrays were immersed twice in xylene for 10 min each. Next, the slides were sequentially immersed in 100%, 95%, 85% and 70% ethanol for 5 min each; immersed in distilled water for 5 min; and then washed twice with PBS for 5 min each. The array slides were incubated with a final developmental 3% H2O2 in PBS (pH = 7.4) at room temperature for 10 min and then rinsed twice in PBS for 5 min each. Antigen retrieval was carried out in 0.01 M sodium citrate buffer(pH = 6.0), the slides were immersed in the buffer for 15 min when it was heated to about 95°C and then cooled down to room temperature before it was washed twice with PBS for 5 min each. The array slides were incubated with blocking antibody for 20–30 min at room temperature followed by a primary antibody (1:50) at room temperature for 1–2 hours. After the array slides were washed twice with PBS, it was incubated with secondary antibody at room temperature for 15–30 minutes and then washed twice again. The DAB reagent was applied at room temperature for 3–5 minutes, and the array slides were washed with distilled water. Finally, the array slides were stained and differentiated in hematoxylin. The array slides were prepared and observed through a regular microscope [11].

Tissue microarrays BR963a was purchased from Alenabio. Detailed information on tissue microarrays FDA800, BR486 and BR963a are available at http://www.alenabio.com/tissue-array/Breast/BR963a.

### Mammalian cells and reagents

The MDA-MB-231, MCF-7, MCF-10A and SK-BR-3 cells were purchased from ATCC. All of the cells were grown at 37°C in 5% CO_2_, 100% relative humidity atmosphere in RPMI 1640 (Hyclon) with 10% FBS(fetal bovine serum). Etoposide was obtained from Peking University Third Hospital. Pifithrin-β (PFT-β) was purchased from MedChem Express (MCE, CHINA). PFT-β was dissolved in dimethyl sulfoxide (DMSO; Sigma-Aldrich; Merck Millipore).

### RNA interference targeting TFDP3

The TFDP3 siRNA1 (5’-CCGACGACAAAUCAGAAUATT-3’) and TFDP3 siRNA2 (5’-GUCUGAACUUCAACAACUUTT-3’) were used for TFDP3 downregulation. The siRNA (5’-TTCTCCGAACGTGTCACGT-3’) unrelated to TFDP3 was used as the control siRNA (Con). The efficiency of RNA interference on TFDP3 expression was determined by Western blotting.

### Plasmids and siRNA transfection

All of the procedures were carried out according to the manufacturer’s protocol. The cells were seeded the day before transfection so that they would be 60–80% confluent at the time of transfection. The NeofectRNAi Transfection reagent (Neofect) was diluted into Opti-MEM medium (Gibco), mixed and then placed at room temperature for 5 min. DNA for transfection was diluted into the same amount of Opti-MEM medium, mixed and then placed at room temperature for 5 min. Diluted DNA and Neofect reagent were mixed and then incubated at room temperature for 15–30 min. The complex was added to the cells in 6-well plates, and the expression was allowed to proceed for 24–72 hours at 37°C. The transfected cells were then analyzed and used for subsequent experiments.

### Flow cytometric analysis

Samples were prepared for detection in a flow cytometer (BD, Accuri C6 Flow Cytometry), and all of the procedures were carried out according to the manufacturer’s protocol. The samples were carefully stirred before loading to avoid cell clusters. Speed for cell loading was slow, and a total of 10000 cells were collected from each tube. The samples were loaded into the flow cytometer as soon as possible after preparation. Samples were kept in the dark to avoid fluorescence quenching.

### Real-time PCR analysis

TransStart Tip Green qPCR SupreMix was purchased from TransGen Biotech, and all of the procedures were carried out according to the manufacturer’s protocol. For TFDP3, the forward primer was 5’-ATGGACGAGAACCAGACCAG-3’ and the reverse primer was 5’-CCCAGACCTTCATGGAAAGA-3’. For GAPDH, the forward primer was 5’-AATGACCCCTTCATTGAC-3’ and the reverse primer was 5’-TCCACGACGTACTCAGCGC-3’. The cycling parameters were 94°C for 15 s, 60°C for 30 s and 72°C for 60 s; 40 cycles were carried out [12].

### Western Blot analysis

At 48h post-transfection, cells were harvested and lysed in lysis buffer for 30 minon ice. The lysates were quantified using BCA assay and mixed with a loading buffer. The supernatant was used for Western blotting. Proteins were separated by SDS-PAGE and transferred onto polyvinylidene fluoride (PVDF) membranes. The membranes were incubated with primary antibodies, including anti-TFDP3(Abcam SH, China), anti-p53(Santa Cruz Biotechnology, CA, USA), anti-LC3(Abcam, SH, China) and anti-GAPDH (Transgen Biotech, BJ, China), followed by incubation with secondary antibodies conjugated to HRP. Signal development was performed with an ECL kit (QIAGEN). Each experiment was performed three times [13].

### Quantification of autophagy

Autophagy was quantified by counting the percentage of cells showing accumulation of GFP-LC3 in vacuoles (GFP-LC3, of a minimum of 100 cells per preparation in three independent experiments) [14,15]. Cells presenting a mostly diffuse distribution of GFP-LC3 in the cytoplasm and nucleus were considered non-autophagic, whereas cells representing several intense punctate GFP-LC3 aggregates with no nuclear GFP-LC3 were classified as autophagic. Cells were fixed with paraformaldehyde (4% w/v) for GFP-LC3 and immunofluorescence assays.

### Statistical analysis

Statistical significance was determined by the two-tailed paired Student’s t-test in all experiments in this study. The data are presented as means ± standard deviation (SD). Values of p<0.05 were considered statistically significant. Asterisks indicate the statistical significance as follows: * p<0.05; ** p<0.01[16].

## Results

### TFDP3 is expressed only in breast cancer tissues, but rarely expressed in other benign lesions of breast

**Fig 1:**
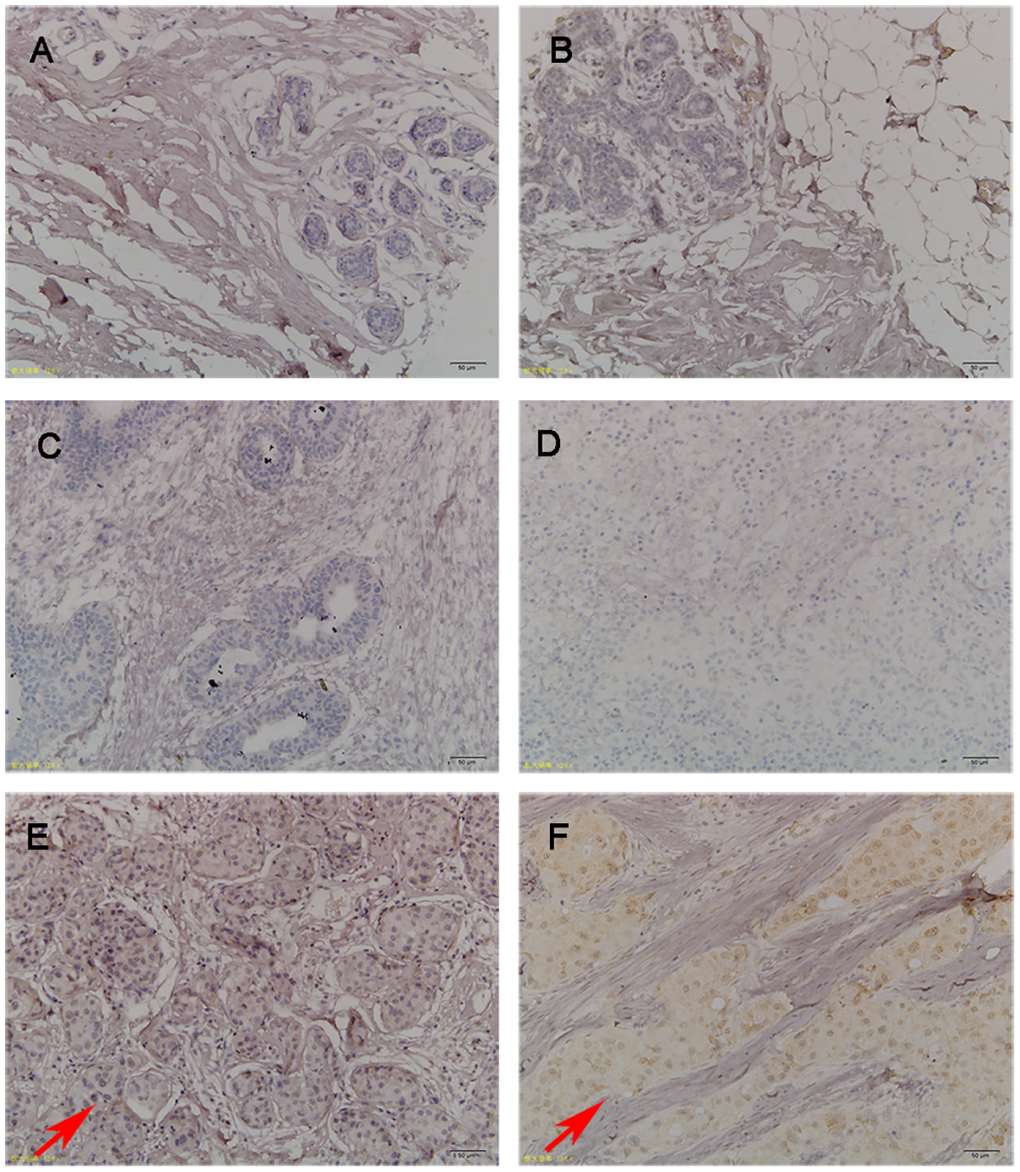
Expressions of TFDP3 in breast benign disease and breast cancer tissues (200×). The figure displays the immunohistochemical staining of: A, Normal breast tissue; B, Adenosis of breast; C, Breast fibroadenoma; D, Plasma cell mastitis; E, Invasive ductal carcinoma; F, Invasive ductal carcinoma. The arrows point to the TFDP3 positive expression cells.

For abnormal breast tissues, TFDP3 is expressed only in breast cancer rather than in other benign breast lesions. We performed an immunohistochemical test on 47 cases of breast diseased tissue sections and 1 case of normal breast tissue section. The results revealed 36 cases of non-specific invasive ductal carcinoma, three cases of plasma cell mastitis, three cases of breast adenoma and breast fibroadenoma, and two cases of invasive lobular carcinoma. Three to five fields of view were randomly selected for each sample (Fig 1). We did not detect TFDP3 expression in normal breast tissue or a variety of benign breast diseases such as plasma cell mastitis and breast adenosis. However, in the breast cancer tissues, various levels of TFDP3 was identified in nearly half of the patients. It mainly expressed in non-special invasive ductal carcinoma tissues and invasive lobular carcinoma tissues. The statistical analysis revealed that TFDP3 is more likely expressed in breast cancers rather than other benign breast lesions (Table 1).

**Table 1:** Expressions of TFDP3 in breast benign disease and breast cancer tissues. The expression of TFDP3 and the correlation between the expression of TFDP3 and the pathologic diagnosis of 47 cases of breast diseases were analyzed by chi-square test and rank-sum test. As TFDP3 only expressed in breast cancer and not in other non-breast cancer diseases, it was concluded that TFDP3 expression only correlates with certain kinds of pathological diagnosis of breast disease.

	N	No. Of tissues with expression of TFDP3					P value
		-	+/-	+	++	+++	
Breast benign disease	9	9	0	0	0	0	0.0433
Breast cancer	38	22	3	8	3	2	

### TFDP3 is expressed in a variety of breast cancer cell lines

**Fig 2:**
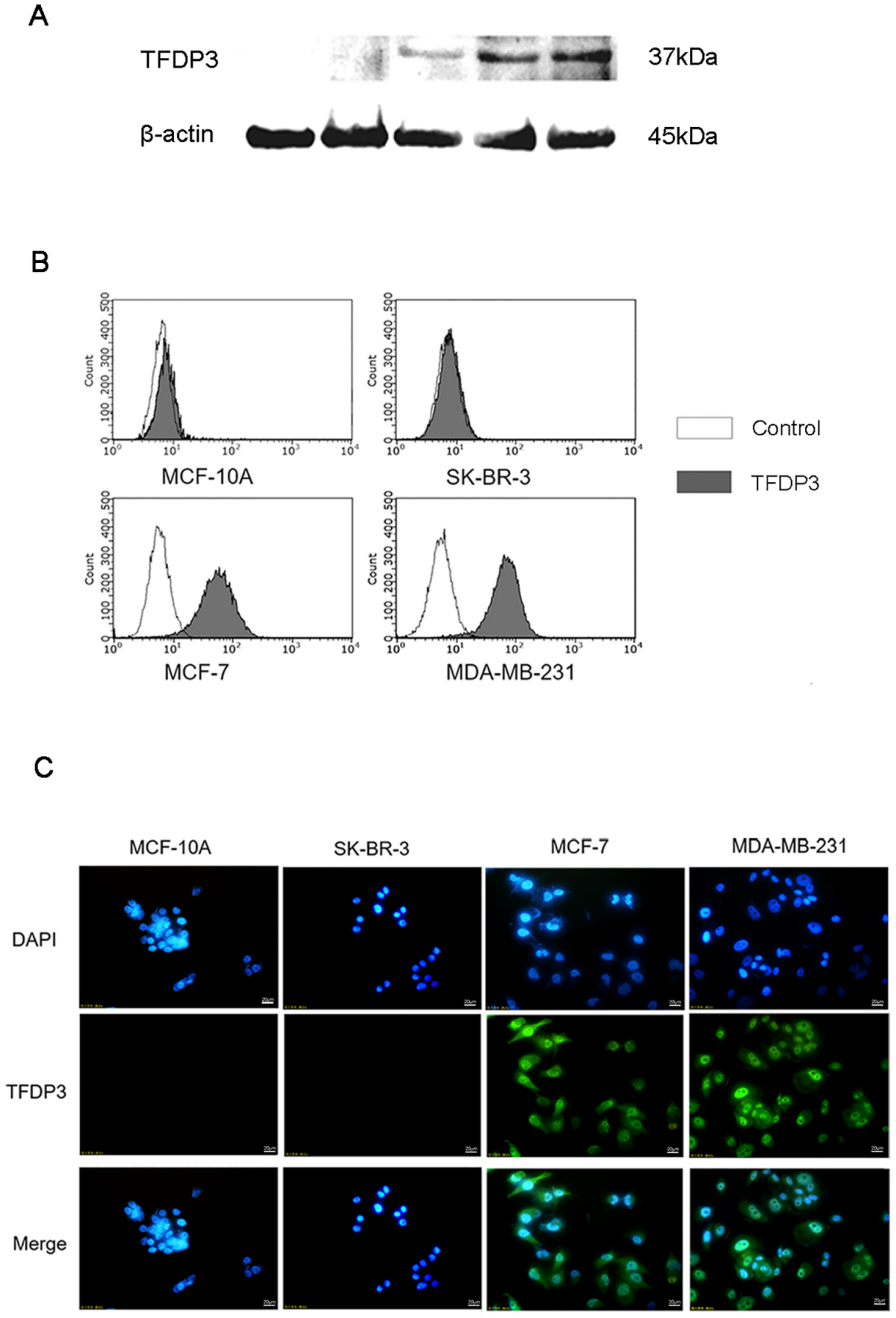
TFDP3 expressions in breast cancer cell lines and testicular tissue. A, Western Blot was used to detect TFDP3 expression in four breast cell lines and testis; B, TFDP3 expression was detected by flow cytometry in both cell lines; C, the subcellular localization of TFDP3 in breast cancer cell line MDA-MB-231 (200×). The expression of TFDP3 in MDA-MB-231 cells was detected both in nuclear and cytoplasm. TFDP3 was green-fluorescence stained and nuclei were stained blue by DAPI.

In order to determine the expression and subcellular localization of TFDP3 in breast cancer, we performed Western Blot analysis on MDA-MB-231, MCF-7, SK-BR-3 and MCF-10A cell lines. To ensure that the total amount of each protein is consistent, BCA protein quantification was executed with testicular tissue set as the positive control. The results showed that TFDP3 protein diverged from each other in MDA-MB-231 and MCF-7 expressions. MDA-MB-231 reached the highest level among all cell lines, while SK-BR-3 and MCF-10A had relatively low or no expression of TFDP3 (Fig 2). Thus, we hypothesized that TFDP3 may play a biological role in the proliferation, apoptosis, migration and metabolism of breast cancer cell line MDA-MB-231.

### The knockdown of TFDP3 by siRNA interference can reduce the resistance of breast cancer cells to etoposide

**Fig 3:**
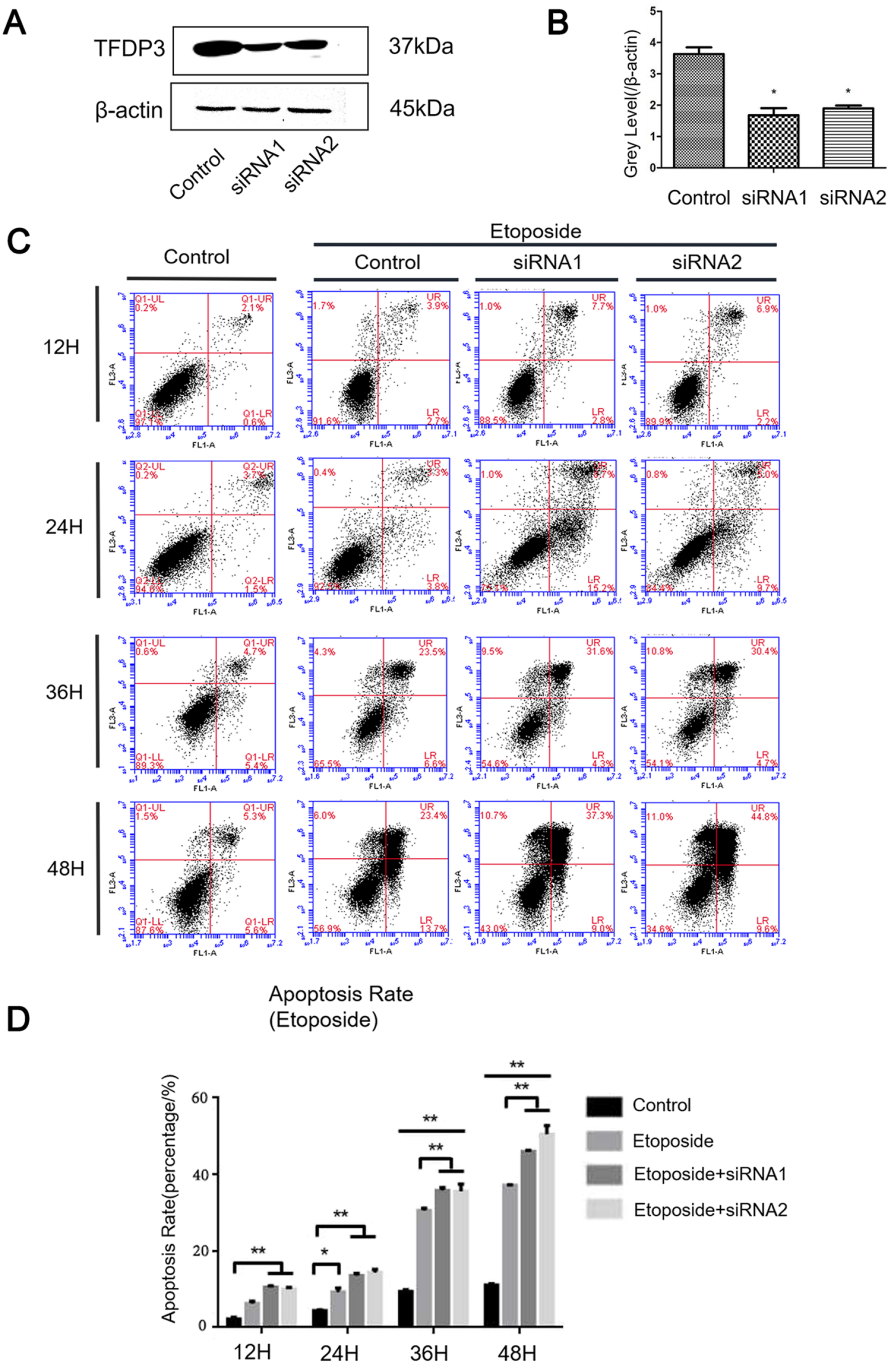
The expression of TFDP3 in siRNA interfered with MDA-MB-231 cancer cell line. A, Western Blot examined knockdown efficiency of TFDP3 by siRNA in MDA-MB-231 cells; B, Image-Pro Plus was used to analyze the gray scale value of Western Blot, and to calculates a relative gray value (TFDP3/β-actin) (n = 6). The results confirmed the expression of TFDP3 in specific siRNA transfected group decreased dramatically; C, Apoptosis rate of siRNA transfected cells with or without 1μM etoposide treatment after 24 hours, 36 hours and 48 hours; Flow cytometry was used examine the apoptosis; D, Histogram of the apoptotic rate (n = 5). Multivariate analysis of variance was performed to analyze significance against the control group, p <0.01.

Previous study screened and selected appropriate siRNA sequences in MDA-MB-231 breast cancer cells which showed satisfactory response to TFDP3 knockdown. We applied siRNA1 and siRNA2 to the establishment of TFDP3 knockdown modeling and NEOFECT was used as the transfection reagent. Then, we deployed Real-time PCR, Western Blot and other methods to validate the knockdown effect of TFDP3 in TFDP3-siRNA transfected MDA-MB-231 cell line (Fig 3A and 3B), independent sample t-test was conducted to examine the knockdown efficacy of these two TFDP3-siRNA. The results of the knockdown efficacy of these two siRNA sequences in MDA-MB-231 cells was significant (p <0.05). So, TFDP3 binding to E2F1 inhibits E2F1-induced apoptosis and increases the tolerance of breast cancer cells to chemotherapeutic agents [17,18]. Therefore, down-regulating TFDP3 expression through transfecting TFDP3-siRNA into MDA-MB-231 cells can increase cell apoptosis. This suggests that TFDP3 may increase the drug resistance of cancer cells and reduce the cell death caused by apoptosis (Fig 3C and 3D). Further, after adding 1μM etoposide drug, the apoptosis rate of breast MDA-MB-231 cancer cells increased over time. Had been transfected with TFDP3-siRNA, the TFDP3 expression rate in the MDA-MB-231 cancer cell dropped and the apoptosis rate increased beyond that of the control group.

### Chemotherapy drug etoposide can induce the expression of LC3 protein in breast cancer cell line MDA-MB-231 and increase the number of autophagosomes

**Fig 4:**
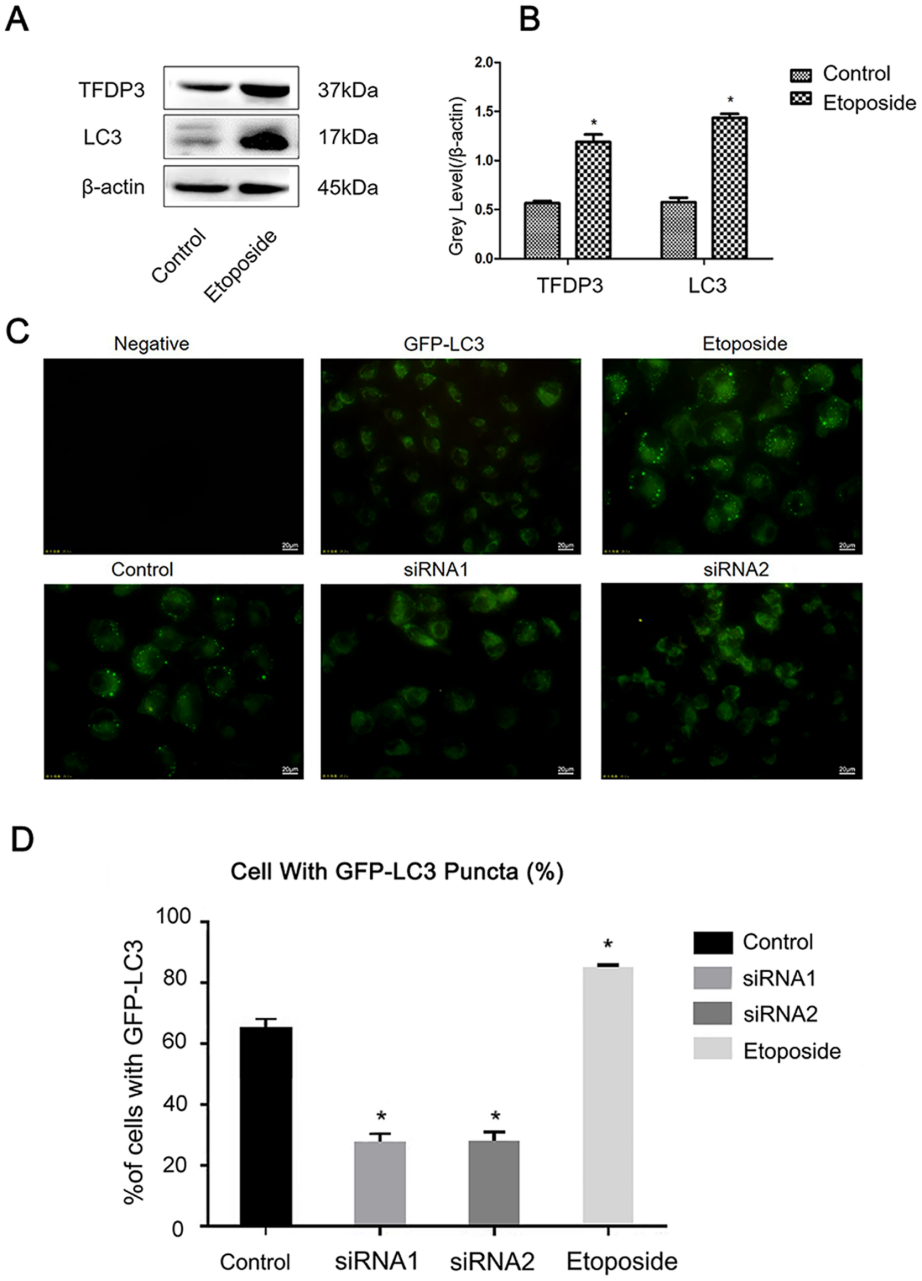
Etoposide on inducing autophagy of breast cancer cell line MDA-MB-231 and the autophagy process after siRNA knocks down TFDP3 expression. A, Expression levels of TFDP3 and LC3 in MDA-MB-231 with or without treatment of etoposide for 24 hours using Western Blot; B, Gray scale statistics for fig 4A. The experiment was repeated for three times and the gray scale of Western Blot stripes was analyzed using Image-Pro Plus software; Then an independent sample T-test was applied to analyze the relative gray values between the GFP-LC3 control group and 1μM etoposide treatment group. For the analysis, the relative gray values (TFDP3/β-actin, LC3/β-actin) were calculated; C, Autophagosomes in all groups, and the green fluorescence of GFP-LC3 were evaluated using fluorescence microscopy (n = 3); D, Percentage of the GFP-LC3 positive cells which exhibited punctate GFP-LC3 fluorescence; All groups were evaluated; cells with >2 autophagosomes per cell were declared as exhibiting an autophagic reaction. An independent sample t-test was used to analyze the number of autophagosomes in the control group, which was significantly higher than the siRNA1 group (p<0.05).

After treatment with 1μM etoposide for 24 hours in breast cancer cell line MDA-MB-231, the expression of TFDP3 and LC3 was up-regulated. The result indicates that etoposide can promote the occurrence of autophagy in cells (Fig 4A and 4B). Then, we introduced GFP-LC3 plasmid into the MDA-MB-231 cell line. Autophagy was measured by assessing the redistribution of GFP-LC3 from a diffuse pattern to cytoplasmic puncta (which are autophagosomes or autophagolysosomes, the membranes of which are decorated by lipidated LC3) [14,15] (Fig 4C and 4D). Non-transfected cells were used as a negative control group, the control group was transfected with GFP-LC3 plasmid, and exogenously introducing this protein made the expression of LC3 at rather high level in the cell. At the same time, the corresponding treatment group consisted of the MDA-MB-231 cell line which was transfected with GFP-LC3 and then treated with 1μM etoposide for 24 hours. The TFDP3 knockdown groups including siRNA1 and siRNA2 respectively showed the numbers of autophagosomes in treated cells, which were transfected siRNA1 or siRNA2 for 24 hours, then introducing GFP-LC3, and overnight stimulation of autophagy with etoposide starting at 48 hours. Using this technology, we found that autophagy would decline in the cells after TFDP3 siRNA transfection.

### TFDP3 affects autophagy by increasing the expression of p53 in breast cancer cell line MDA-MB-231

**Fig 5:**
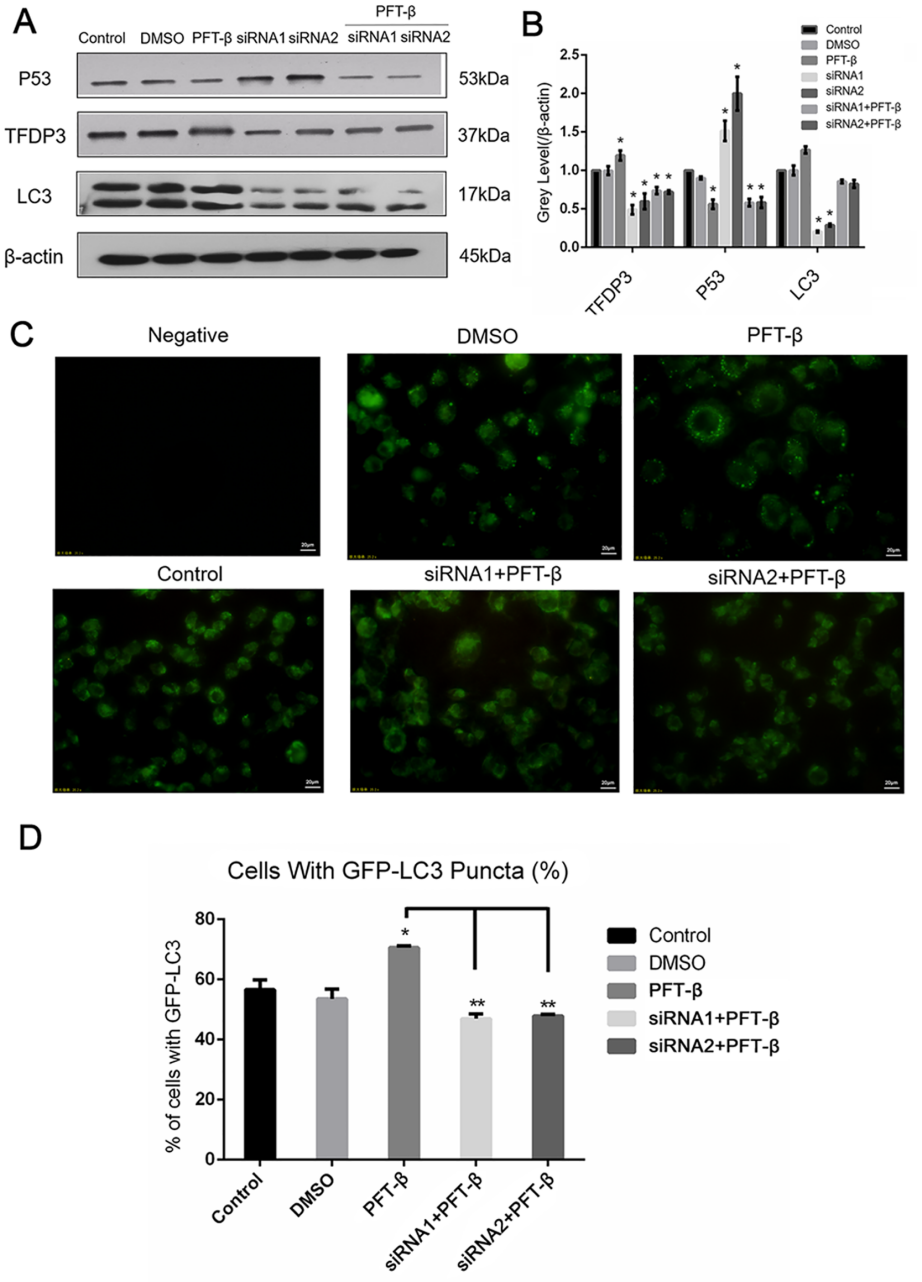
TFDP3 affects the autophagy by increasing the expression of p53 in breast cancer cell line MDA-MB-231. A, Western Blot analysis of TFDP3, p53 and LC3 in whole cell lysates of MDA-MB-231 transfected and treated with indicated plasmids and reagents. Cells in the treatment group were incubated with 20 μM PFT-beta for 24 hours; B, Gray value statistics of the fig 5A. The relative gray values (TFDP3/ beta-actin, p53/ beta-actin, LC3/ beta-actin) were calculated. Data represent the mean ±SD of at least three independent experiments (*, P≤0.05); The experiment was repeated 3 times; C, Fluorescence microscopy pictures of MDA-MB-231 with or without TFDP3 downregulation transfected with GFP-LC3 plasmid or treated with 20 μM PFT-beta; D, Calculated number of autophagosome in cells described in fig 5C. In each group, five fields were randomly selected for calculation and statistical analysis. The data represents the mean ±SD (*, P≤0.05).

To explore how TFDP3 affects autophagy in breast cancer, we transfected the breast cancer cell line MDA-MB-231 with TFDP3-siRNA. In the treatment group, the cell line MDA-MB-231 was treated with 1μM etoposide for 24 hours. After downregulating the expression of TFDP3, we found that the expression of p53 also changed. We used two siRNAs to knock down TFDP3 and quantified that autophagy of marker protein LC3 decreased while p53 expression was raised (Fig 5A and 5B). Current research also reports inhibiting p53 results in a maximum level of autophagy in cells. So, we hypothesize that TFDP3 could regulate the autophagy in breast cancer cells by affecting the expression of p53 directly or indirectly. To verify this, we incubated TFDP3 knockdown cells using indicated siRNA with p53 inhibitor PFT-β for 24 hours then examined the autophagy marker LC3 using Western Blot and observed the autophagosomes by confocal microscopy. The activation of autophagy increased in TFDP3 knockdown groups after both the expression of LC3 and numbers of GFP-LC3 puncta were up-regulated (Fig 5C and 5D). Meanwhile, in the treatment groups (siRNA+PFT-β groups), TFDP3 expression decreased, the expression of p53 in the cells increased—so that LC3 decreased—and intracellular autophagy weakened. Thus, TFDP3 affects autophagy in MDA-MB-231 cells through p53 pathway, resulting in the reduction of the release of chemotherapy drug etoposide from cancer cells. Moreover, the apoptosis of breast cancer cells increased (as showed in the previous results). These results support our hypothesis that knocking down TFDP3 in the breast cancer cell MDA-MB-231 can reduce the tolerance to the chemotherapy drug etoposide. However, the details of this mechanism need to be further investigated.

## Discussion

TFDP3 is a cancer/testis antigen and, a member of the DP family of transcription factors which is encoded by a gene located on the long arm of chromosome X at position Xq26.2[19]. In this study, we found that TFDP3 was widely expressed in breast cancer cells and, identified its subcellular localization in both the nucleus and the cytoplasm. The different localization is related to the status and biological function of the cancer-testis antigen in the cell signaling pathway [17]. Thus, the intracellular localization of TFDP3 in both the nucleus and the cytoplasmic of breast cancer cells indicates that TFDP3 may play a biological role in cell proliferation, apoptosis, migration and metabolism.

The results of the flow cytometry test show that, following etoposide treatment, the apoptosis rate of breast cancer cell line MDA-MB-231 significantly increases after TFDP3-siRNA transfection. This suggests that less expression of TFDP3 in breast cancer cells might up-regulate the sensitivity to chemotherapy drugs and eventually lead to cell apoptosis. Moreover, it can be inferred that TFDP3 also plays an important role in the process of drug resistance of breast cancer, which can prevent chemotherapy-induced apoptosis of tumor cells. Thus, TFDP3 can compete with endogenous TFDP1 for E2F1 binding to form inactive heterodimers. As a non-DNA-binding TFDP3/E2F1 complex, it can inhibit the transcriptional activity of the downstream gene. Hence, the apoptosis of cancer cells is inhibited because the E2F1-activated apoptotic signaling pathway was blocked [18].

In this study, we also showed that TFDP3 was linked to cell autophagy. Autophagy is not only an intracellular process for maintaining cell metabolism, but also a survival mechanism which could improve the resilience of cells to environmental stress. In fact, it is one of the most crucial mechanisms of cell-damage repair in the process of tumor cell resistance. As it is the first identified programmed cell death process, the regulatory mechanism of apoptosis has been studied thoroughly [20]. Current studies reveal that apoptosis and autophagy dysfunction are associated with cancer development and chemotherapy resistance [21]. Further, cell autophagy has a dual effect for cancer cells. In the early stage of tumorigenesis, autophagy can inhibit the process by eliminating tumorigenic metabolites, suppressing chronic inflammation, and regulating oncogene-induced senescence. Similarly, in the late stage of the cancer, autophagy can induce the survival of cancer cells and promote the growth of cancer cells in adverse environments [22,23]. In the current research, cancer cells repairing chemotherapy-caused DNA and cell damage through autophagy has been considered as an important mechanism of cancer cell resistance to chemotherapy drugs [24–26]. Meanwhile, tumor protein p53 can inhibit tumorigenesis and regulate the activities of intracellular apoptosis and autophagy, so that cancer cells can survive even under stressful conditions. The protective mechanism of p53 includes maintaining the stability of the intracellular genome and the metabolic homeostasis, and reducing the possibility of cancer occurring in healthy organs. However, some abnormal cells may also utilize the protective mechanism of p53 to develop into cancer cells, which then gain chemotherapy resistance [6,27,28]. We conclude that TFDP3 can initiate autophagy by influencing the expression of p53 to produce resistance to cancer cells, to resist the effects of chemotherapeutic drugs, and to repair DNA damage.

The results show that, under the treatment of chemotherapy drugs, the drug resistance of cancer cells through autophagy activation is closely related to the expression level of TFDP3. Downregulation of the expression of TFDP3 through the application of siRNA can inhibit the expression of the autophagy-related protein, LC3, and can reduce the number of autophagosomes in cancer cells. Based on bioinformatics analysis, TFDP3 shares a high degree of sequence homology with TFDP1, both of which have the domain to bind to E2F family members to form heterodimers. However, the amino acid residue sequence of each DNA binding region is different, which results in E2Fs/TFDP3 inactivating DNA-binding while E2Fs/TFDP1 promotes DNA-binding. Our study suggests that in the process of autophagy, the different function of E2F1 could be due to the different component of TFDPs in E2F1/TFDPs heterodimers. In normal cells, E2F1 binds to TFDP1 to form a complex when the cells are damaged or stressed. This E2F1/TFDP1 complex binds to the TTT(C/G)GCGC(C/G) sequence to trigger downstream transcription and expression of apoptosis-related genes, and induce cells entry into the apoptosis process. However, in various cancer cells, the expression of TFDP3 molecule is much higher. TFDP3 could compete with TFDP1 for E2F1 binding, and the E2F1/TFDP3 heterodimer may not activate the downstream autophagy-related gene transcription and expression; Therefore, the cell autophagy will be blocked. In the treatment of malignant tumors, the demethylation in the promoter region of TFDP3 could continuously upregulate the expression of TFDP3 in cancer cells. That cancer cells can repair the damage of DNA and organelles caused by chemotherapy through autophagy activation is an important prognostic significance for tumor recurrence and drug resistance.

Consequently, cancer-testis antigen TFDP3 plays a very significant regulatory role in the emergence of drug resistance in breast cancer and other solid malignant tumor cells. The application of siRNA interference technology could improve sensitivity of TFDP3 positively in breast cancer cells to chemotherapy in vitro by down-regulating the expression of TFDP3, so enhancing the effect of chemotherapy and offering important insights into clinical therapy via the integration of chemotherapy with immunotherapy.

## Conclusion

This study explored the function of TFDP3 in cancer cell autophagy combining with E2F1. The results show that TFDP3 promoted the chemotherapy-induced cell autophagy process in breast cancer cells. By down-regulating the expression of TFDP3, the formation of autophagosome and the expression of LC3, the key protein of autophagy, is reduced following chemotherapy treatment. This study provides an important experimental basis for reducing the autophagy-associated tumor cell resistance and improving the comprehensive therapeutic effect of cancers by regulating the expression and activity of TFDP3. It also lays the foundation for exploring the structure and function of E2F/TFDP complex in gene transcription and regulation.

The results of our study show that the cancer/testis antigen TFDP3 molecule is a potential tumor antigen marker; and, also, one of the important transcriptional regulatory molecules of chemotherapy drug resistance. It participates in tumor drug resistance through both antagonizing E2F1-induced apoptosis and, the induction of autophagy in tumor cells. Our conclusions suggest that TFDP3-associated gene target therapy may be able to reduce tumor drug resistance and, thereby, prolong the patients’ survival. Furthermore, TFDP3 target therapy may also work with immunotherapy as a part of combined treatment method for cancer.

## Supporting information

S1 File The raw data of some figures and tables in the paper. Folder A: The uncropped Western blot images of Figs 2A, 3A, 4A and 5A.
(ZIP)
