# Supplementary material for: TFDP3 regulates the apoptosis and autophagy in breast cancer cell line MDA-MB-231
Source: PLoS One. 2018 Sep 20;13(9):e0203833. doi: 10.1371/journal.pone.0203833 (PMC6147432; doi:10.1371/journal.pone.0203833)
Supplement: S1 File — Folder A: The uncropped Western blot images of Figs 2A, 3A, 4A and 5A. (ZIP) [file pone.0203833.s001.zip › Folder A/Supporting information.docx]

# Supporting information

**S1 File. The raw data of some figures and tables in the paper**

It is the catalog of files in the zip file below:

Folder A: The uncropped Western blot images of Fig 2A, Fig 3A, Fig 4A, and Fig 5A.
