# Supplementary figures and images for: TFDP3 regulates the apoptosis and autophagy in breast cancer cell line MDA-MB-231
Source: PLoS One. 2018 Sep 20;13(9):e0203833. doi: 10.1371/journal.pone.0203833 (PMC6147432; doi:10.1371/journal.pone.0203833)

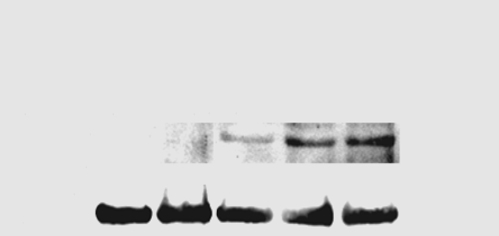

Supplement: S1 File — Folder A: The uncropped Western blot images of Figs 2A, 3A, 4A and 5A. (ZIP) [file pone.0203833.s001.zip › Folder A/Raw data Figure 2/Raw data Figure 2A.jpg]

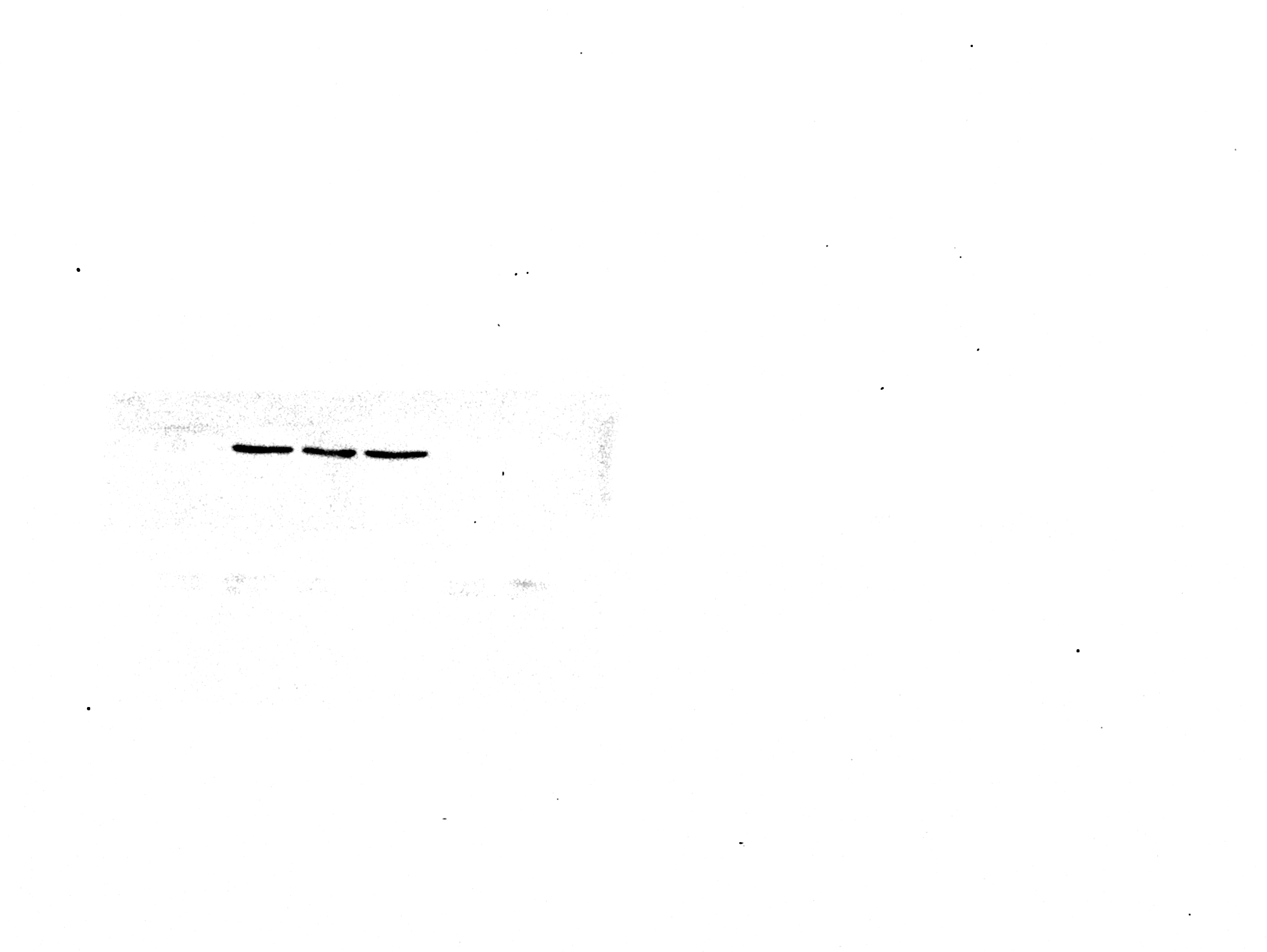

Supplement: S1 File — Folder A: The uncropped Western blot images of Figs 2A, 3A, 4A and 5A. (ZIP) [file pone.0203833.s001.zip › Folder A/Raw data Figure 3/Raw data Figure 3A-beta action.tif]

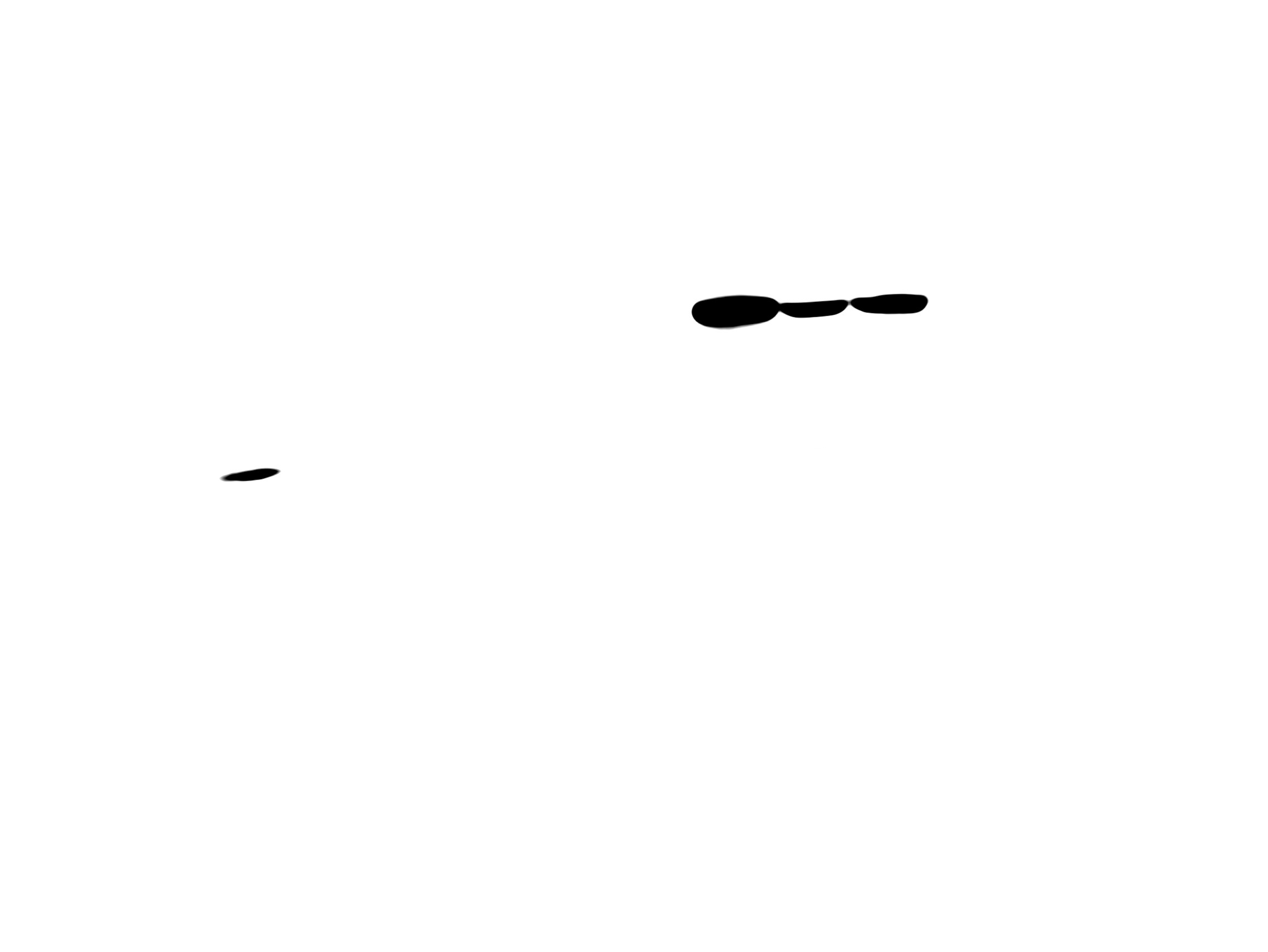

Supplement: S1 File — Folder A: The uncropped Western blot images of Figs 2A, 3A, 4A and 5A. (ZIP) [file pone.0203833.s001.zip › Folder A/Raw data Figure 3/Raw data Figure 3A-TFDP3.tif]

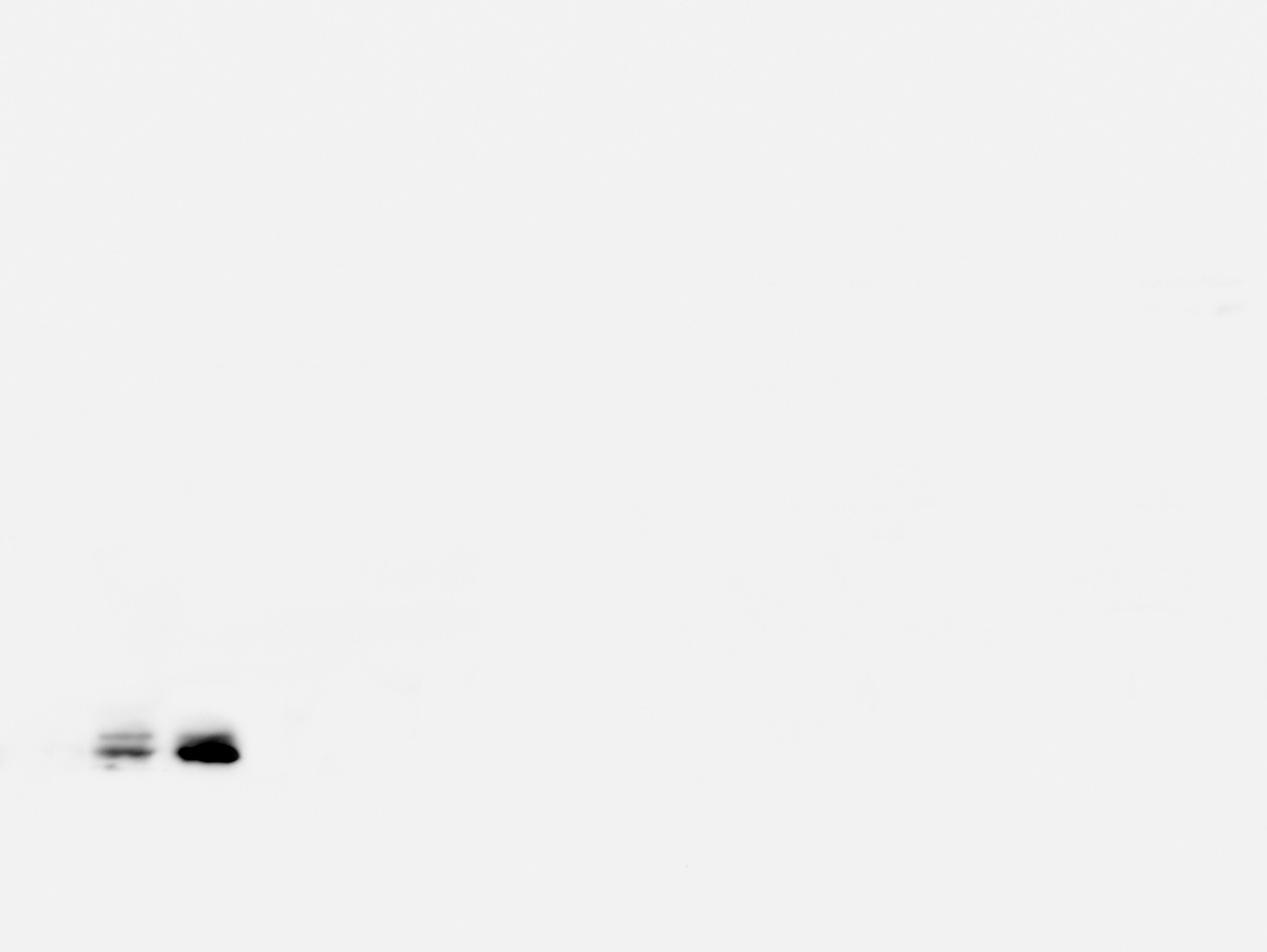

Supplement: S1 File — Folder A: The uncropped Western blot images of Figs 2A, 3A, 4A and 5A. (ZIP) [file pone.0203833.s001.zip › Folder A/Raw data Figure 4/Raw data Figure 4A-LC3.jpg]

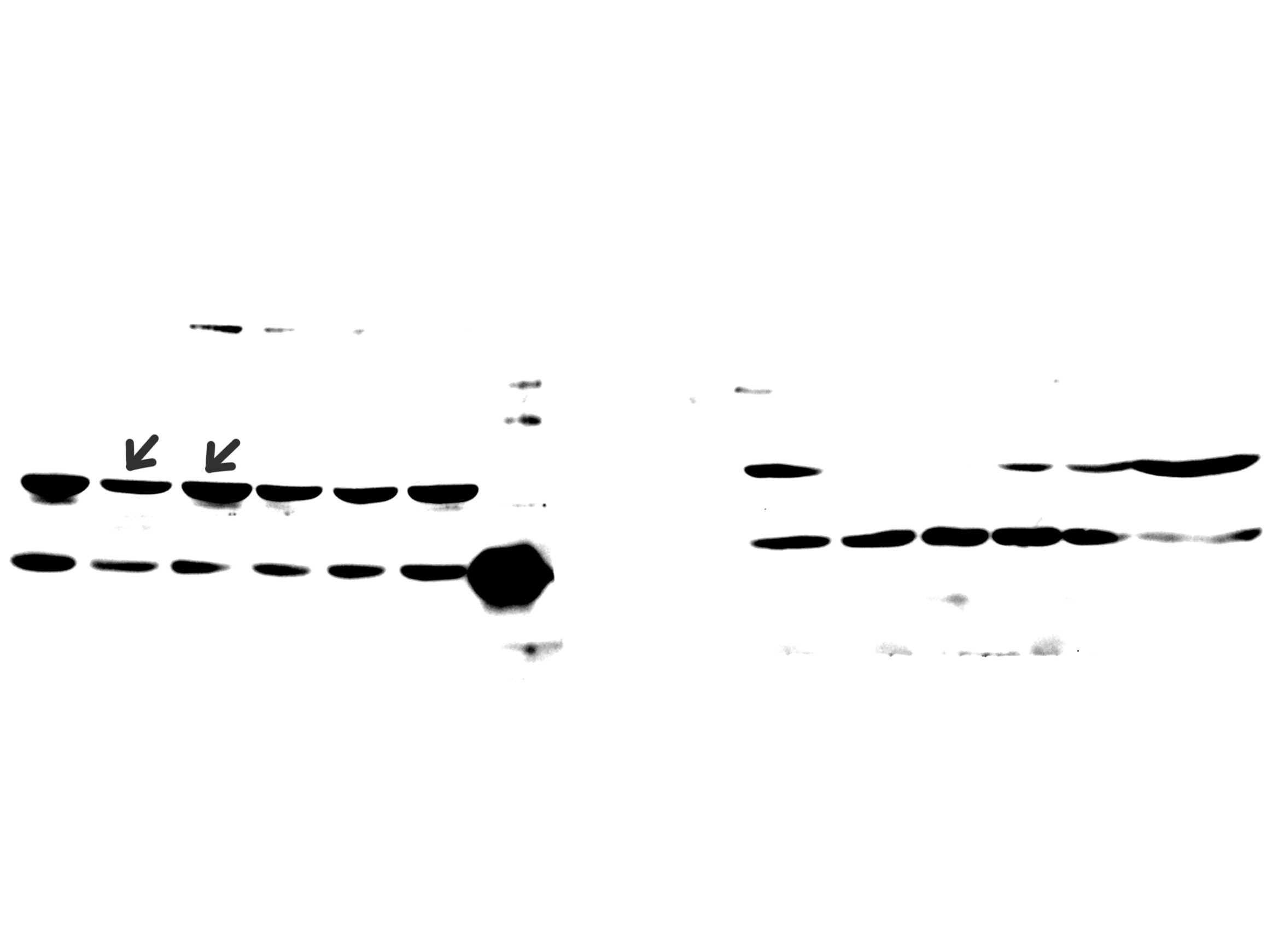

Supplement: S1 File — Folder A: The uncropped Western blot images of Figs 2A, 3A, 4A and 5A. (ZIP) [file pone.0203833.s001.zip › Folder A/Raw data Figure 4/Raw data Figure 4A-TFDP3 beta-actin.jpg]

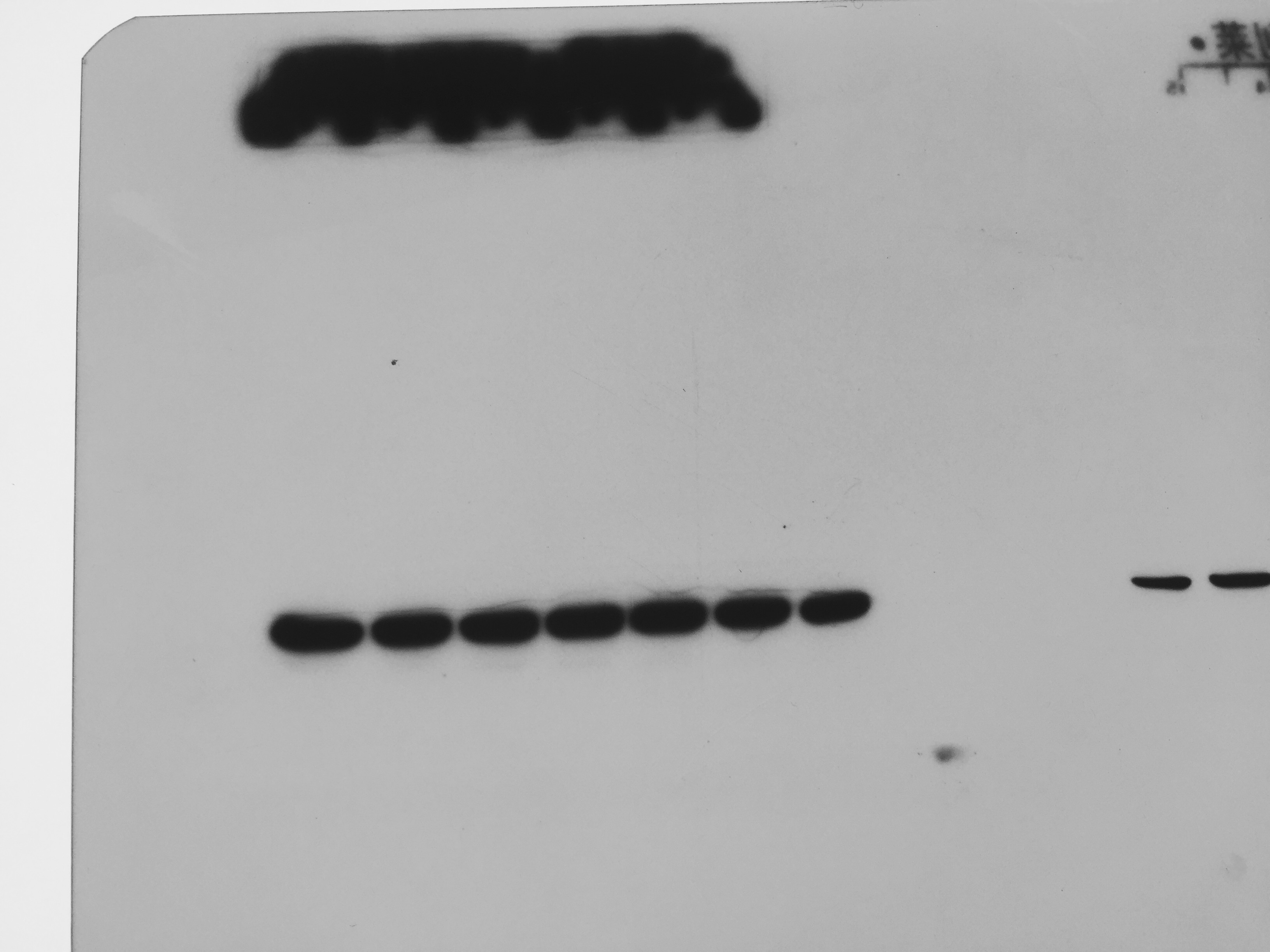

Supplement: S1 File — Folder A: The uncropped Western blot images of Figs 2A, 3A, 4A and 5A. (ZIP) [file pone.0203833.s001.zip › Folder A/Raw data Figure 5/Raw data Figure 5A-beta actin.jpg]

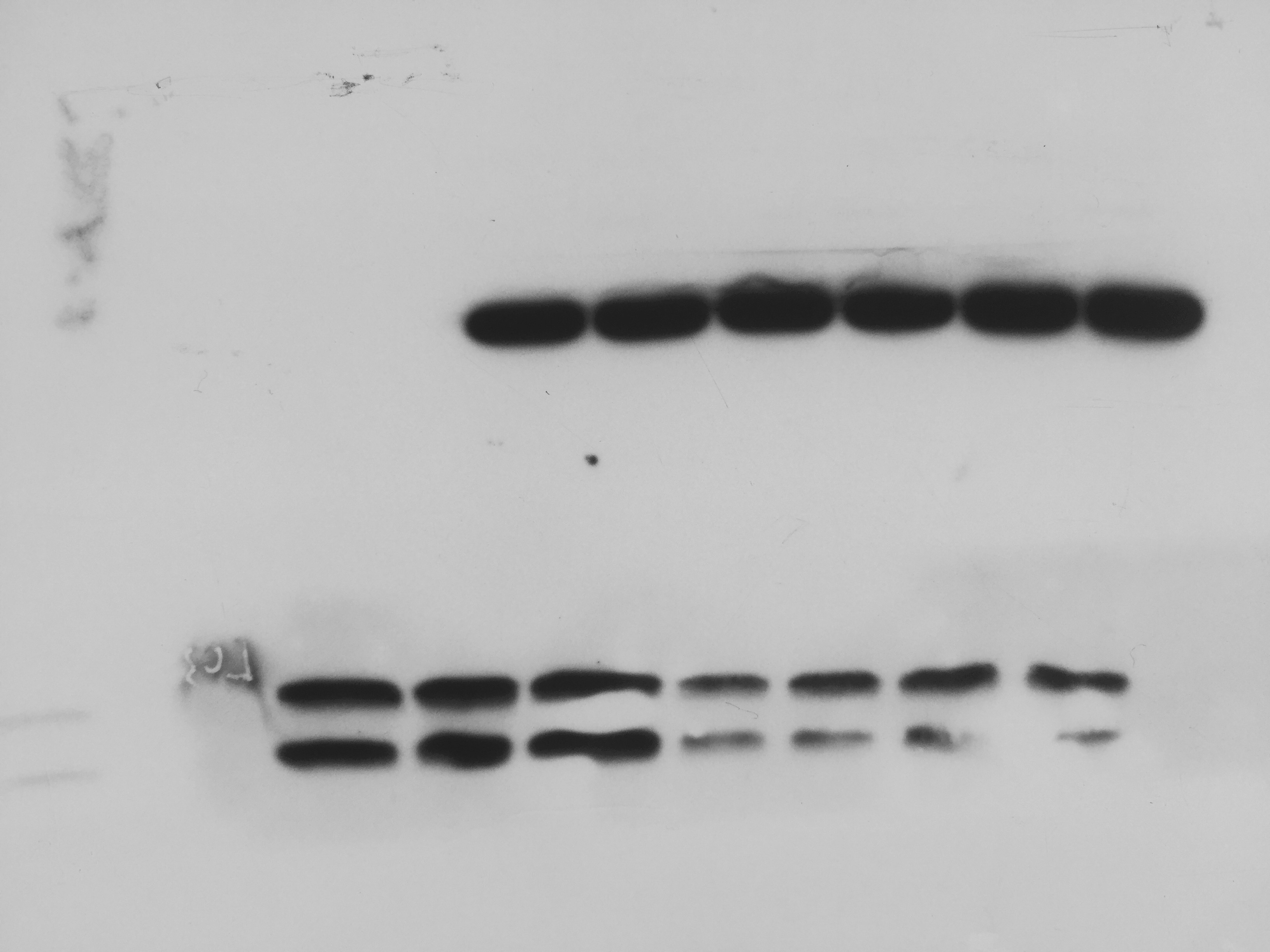

Supplement: S1 File — Folder A: The uncropped Western blot images of Figs 2A, 3A, 4A and 5A. (ZIP) [file pone.0203833.s001.zip › Folder A/Raw data Figure 5/Raw data Figure 5A-LC3.jpg]

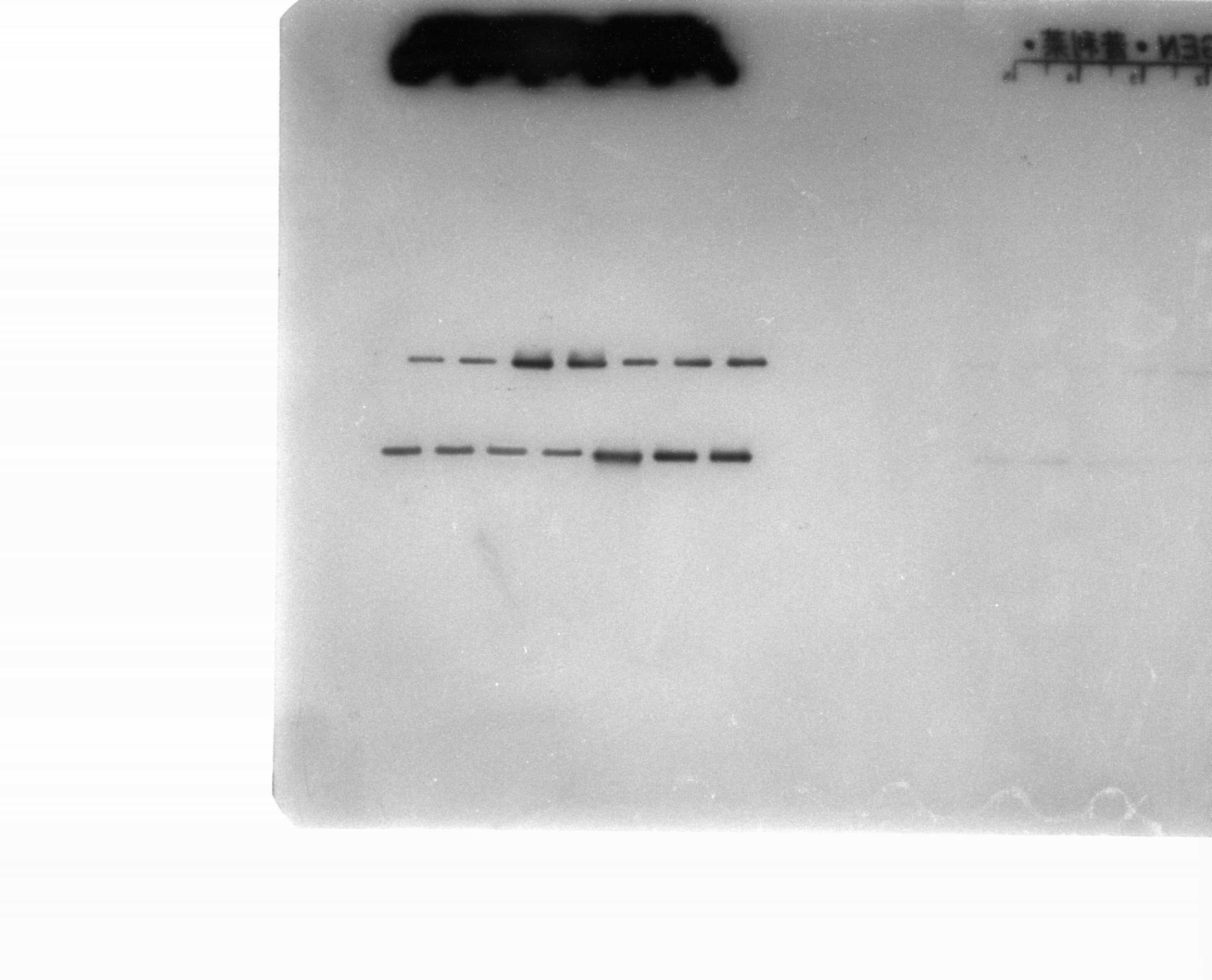

Supplement: S1 File — Folder A: The uncropped Western blot images of Figs 2A, 3A, 4A and 5A. (ZIP) [file pone.0203833.s001.zip › Folder A/Raw data Figure 5/Raw data Figure 5A-p53 TFDP3.tif]
